# Antimicrobial resistance and antibiotic consumption in intensive care units, Switzerland, 2009 to 2018

**DOI:** 10.2807/1560-7917.ES.2021.26.46.2001537

**Published:** 2021-11-18

**Authors:** Stefanie Barnsteiner, Florent Baty, Werner C Albrich, Baharak Babouee Flury, Michael Gasser, Catherine Plüss-Suard, Matthias Schlegel, Andreas Kronenberg, Philipp Kohler, on behalf of the Swiss Centre for Antibiotic Resistance (ANRESIS)

**Affiliations:** 1 Division of Infectious Diseases and Hospital Epidemiology, Cantonal Hospital St. Gallen, St. Gallen, Switzerland; 2 Lung Center, Cantonal Hospital St. Gallen, St. Gallen, Switzerland; 3 Medical Research Center, Cantonal Hospital St. Gallen, St. Gallen, Switzerland; 4 Institute for Infectious Diseases, University of Bern, Bern, Switzerland

**Keywords:** Carbapenem resistance, Carbapenem-resistant Enterobacterales, CRE, Gram-negative bacteria, antibiotic consumption, intensive care, ICU, Switzerland

## Abstract

**Background:**

Intensive care units (ICU) constitute a high-risk setting for antimicrobial resistance (AMR).

**Aim:**

We aimed to describe secular AMR trends including meticillin-resistant *Staphylococcus aureus* (MRSA), glycopeptide-resistant *enterococci* (GRE), extended-spectrum cephalosporin-resistant *Escherichia coli* (ESCR-EC) and *Klebsiella pneumoniae* (ESCR-KP), carbapenem-resistant *Enterobacterales* (CRE) and *Pseudomonas aeruginosa* (CRPA) from Swiss ICU. We assessed time trends of antibiotic consumption and identified factors associated with CRE and CRPA.

**Methods:**

We analysed patient isolate and antibiotic consumption data of Swiss ICU sent to the Swiss Centre for Antibiotic Resistance (2009–2018). Time trends were assessed using linear logistic regression; a mixed-effects logistic regression was used to identify factors associated with CRE and CRPA.

**Results:**

Among 52 ICU, MRSA decreased from 14% to 6% (p = 0.005; n = 6,465); GRE increased from 1% to 3% (p = 0.011; n = 4,776). ESCR-EC and ESCR-KP increased from 7% to 15% (p < 0.001, n = 10,648) and 5% to 11% (p = 0.002; n = 4,052), respectively. CRE, mostly *Enterobacter* spp., increased from 1% to 5% (p = 0.008; n = 17,987); CRPA remained stable at 27% (p = 0.759; n = 4,185). Antibiotic consumption in 58 ICU increased from 2009 to 2013 (82.5 to 97.4 defined daily doses (DDD)/100 bed-days) and declined until 2018 (78.3 DDD/100 bed-days). Total institutional antibiotic consumption was associated with detection of CRE in multivariable analysis (odds ratio per DDD: 1.01; 95% confidence interval: 1.0–1.02; p = 0.004).

**Discussion:**

In Swiss ICU, antibiotic-resistant *Enterobacterales* have been steadily increasing over the last decade. The emergence of CRE, associated with institutional antibiotic consumption, is of particular concern and calls for reinforced surveillance and antibiotic stewardship in this setting.

## Introduction

Intensive care units (ICU) are considered high-risk settings for antimicrobial resistance (AMR) [1]. This can be explained by an increased risk for nosocomial infections in ICU patients who have underlying comorbidities and frequently use of medical devices, but also by potentially increased exposure to resistant organisms where there is high patient turnover and by the large number of transmission opportunities [2]. Another particularity of the ICU setting is the extensive use of broad-spectrum antibiotics, which is considered to be one of the main drivers for the development and dissemination of AMR, in particular for resistant Gram-negative pathogens [3]. Indeed, several studies have reported an association between carbapenem consumption and the emergence of carbapenem-resistant *Enterobacterales* (CRE) and *Pseudomonas aeruginosa* (CRPA) [4,5]. The CRE and CRPA are pathogens of particular interest, as both have been classified by the World Health Organisation (WHO) as critical pathogens regarding the need for new antibiotic substances [6].

Surveillance is considered a cornerstone in the fight against AMR [7]. Specific surveillance data from AMR in Swiss ICU have not been published so far, although outbreaks caused by resistant pathogens have been reported [8]. Data from the Swiss Centre for Antibiotic Resistance (ANRESIS) for non-ICU settings have shown a steady rise of AMR among Gram-negative pathogens between 2007 and 2017, while meticillin-resistant *Staphylococcus aureus* (MRSA) are declining [9,10]. Antibiotic consumption in Swiss ICU has been shown to be somewhat lower than in other European countries; however, these data were collected between 2004 and 2008 [11].

In this study, we describe secular trends of AMR and antibiotic consumption from Swiss ICU using surveillance data from ANRESIS. For CRE and CRPA, we sought to identify patient-level and institutional factors associated with resistance, including institutional antibiotic consumption. We also assessed the representativeness of ANRESIS for the ICU setting in Switzerland.

## Methods

### Data source

This laboratory-based surveillance study was conducted using antimicrobial resistance and antibiotic consumption data from ANRESIS (www.anresis.ch). ANRESIS, led by the Institute for Infectious Diseases at the University of Bern, is the national centre for surveillance of antibiotic resistance and antibiotic consumption and is supported by the Swiss Federal Office of Public Health. Antimicrobial susceptibility testing is performed at local laboratories according to guidelines from the Clinical and Laboratory Standards Institute (CLSI) or the European Committee on Antimicrobial Susceptibility Testing (EUCAST) [12,13]. All laboratories are accredited by Swiss authorities and participate in at least one external quality programme of either the National External Quality Assessment Service (https://ukneqas.org.uk) or the Swiss quality control programme of the Institute for Medical Microbiology, University of Zurich (www.imm.uzh.ch/de/services/qc.html). 

### Representativeness of ANRESIS

ANRESIS continuously collects routine antibiotic resistance data (along with basic patient information including age category, sex and sample location) from 33 laboratories distributed homogeneously all over Switzerland and representing 89% of all hospital samples in 2020. Antibiotic consumption data are collected once yearly from around 70 hospital pharmacies across Switzerland. For the analysis of AMR in ICU, we assessed the representativeness of ANRESIS for ICU registered by the Swiss Society for Intensive Care (www.sgi-ssmi.ch/de/zertifizierte-is.html). Because several small-size ICU have been closed or have been incorporated into larger institutions over the last decade, representativeness was only assessed for the year 2018. This was done by calculating the percentage of ICU beds represented in ANRESIS among all registered ICU beds in Switzerland, stratified by administrative subdivisions (i.e. cantons) speaking mostly French or Italian (cantons of Fribourg, Geneva, Jura, Neuchâtel, Ticino, Valais and Vaud; henceforth referred to as south-west) or German (all other cantons; henceforth referred to as east). In order to detect differences in microbiological sampling frequencies between geographical regions, we calculated the sampling density, defined as the number of microbiological samples per represented ICU bed.

### Inclusion criteria for antimicrobial resistance and antibiotic consumption data

For the analysis of AMR, we extracted microbiological isolates with the label 'intensive care' sent to ANRESIS between January 2009 and December 2018 from patients 15 years and older. Although data were anonymous, every patient possesses a unique identity number within every institution, which was used to exclude repeat isolates (i.e. same pathogen and resistance profile) from the same patient in a specific calendar year. If patients contributed both resistant and susceptible isolates (per species) in the same year, only the resistant isolate was included. Therefore, each patient contributed only one sample per species, institution and year. Bacterial isolates from non-specified swabs (presumably rectal swabs taken at time of admission) were excluded in order to minimise the impact of patients colonised with resistant pathogens before ICU admission.

For the analysis of antibiotic consumption, we extracted consumption data for the same time period (2009 to 2018) from those ICU where these data were available. Of note, antibiotic consumption data were only available on the institutional level (per year) and not on the patient level.

For the analysis of risk factors for CRE and CRPA, we included only data from years where institutions provided AMR data along with corresponding antibiotic consumption data.

### Definitions for antimicrobial resistance

Resistance to a particular substance was defined as either an intermediate or a resistant phenotype in susceptibility testing according to the individual laboratories. Group resistance (e.g. glycopeptide, carbapenem, aminoglycoside or quinolone resistance) was defined as resistance or intermediate susceptibility to at least one antibiotic of the respective group; extended-spectrum cephalosporin-resistance was defined as resistance or intermediate susceptibility to at least one third or fourth generation cephalosporin.

Among Gram-positive bacteria, we analysed MRSA and glycopeptide-resistant *Enterococcus faecalis/faecium* (GRE). Non-multidrug-resistant MRSA was used as a surrogate for community-associated MRSA (caMRSA), defined as being susceptible to at least three of the following agents: ciprofloxacin, clindamycin, tetracycline and trimethoprim-sulfamethoxazole [10]. The following Gram-negative pathogens were analysed: extended-spectrum cephalosporin-resistant *Escherichia coli* (ESCR-EC) and *Klebsiella pneumoniae* (ESCR-KP), CRE (of note, species with intrinsically low activity against imipenem such as *Proteus* spp., *Providencia* spp. or *Morganella* spp. were excluded), CRPA (of note, only resistance to imipenem or meropenem was considered), carbapenem-resistant *Acinetobacter baumannii* complex (CRAB) and trimethoprim-sulfamethoxazole-resistant *Stenotrophomonas maltophilia* (TSRSM).

### Data analysis and statistics

We expressed AMR as a percentage of all isolates and consumption of systemic antibiotics as defined daily doses (DDD) per 100 bed-days (BD), compliant with the World Health Organization (WHO) definition from 2019 [14]. Categorical variables were reported as frequencies and proportions, continuous variables as median with interquartile range (IQR). For dichotomous variables, we used chi-squared or Fisher's exact test, as appropriate. For continuous variables, we used the Wilcoxon rank-sum test. For each type of resistance, the proportion of pathogens with the resistance trait (as percentage of the total number of isolates reported and tested for the respective key antibiotic) was analysed over time. Linear regression analysis was used to estimate temporal trends, using resistance or antibiotic consumption as dependent and year of surveillance as independent variable.

For the analysis of risk factors for CRE and CRPA, we performed a mixed model with ICU as random effect, considering the hierarchical data structure. Patient-level variables included age group (< 60, 60–75 and > 75 years), sex and site of sample collection (blood, respiratory, urogenital or other). Institutional variables included geographical region (south-west vs east), hospital type (university vs non-university hospitals) and total annual antibiotic consumption. All co-variables were entered into multivariable analysis. Two-sided p values ≤ 0.05 were considered statistically significant. All statistical analyses were performed using R statistical software version 3.5.2 (R Foundation, Vienna, Austria).

### Sensitivity analyses

Sensitivity analyses were performed for the assessment of temporal trends for AMR and antibiotic consumption, including only those ICU that provided data for at least 8 years between 2009 and 2018, in order to eliminate potential selection bias.

### Ethical statement

Because ANRESIS contains routinely collected anonymised surveillance data, ethical consent was not required according to the Swiss law for research on human beings.

## Results

### Representativeness of included intensive care units

Over the 10-year period, we included 52 and 58 ICU for the analysis of AMR and antibiotic consumption, respectively (for an overview see Supplementary Figure S1). By 2018, 11 of 52 ICU did no longer exist, resulting in 41 ICU represented in our database for the analysis of AMR. Of these 41 ICU (with a median bed number of nine), 17 were located in south-western, and 24 in eastern Switzerland; the median bed number was 39 (range: 34–64) for the five university and eight (range: 6–42) for the 36 non-university ICU (listed in Supplementary Table S1).

**Figure 1:**
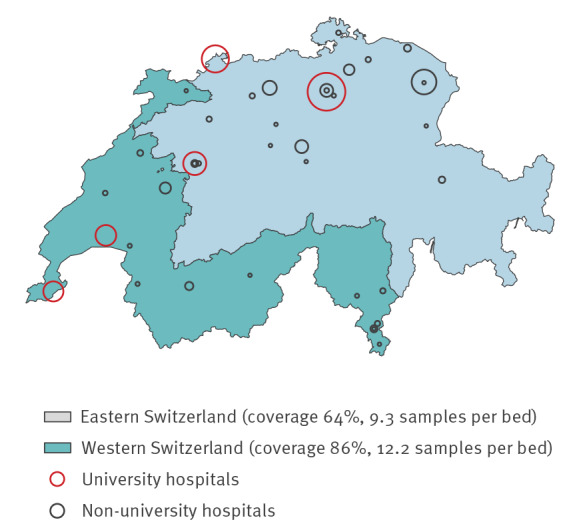
Intensive care units represented in the analysis of antimicrobial resistance, Switzerland, 2009–2018 (n = 41)

The 41 ICU covered 55% of the 74 registered ICU in Switzerland for the year 2018, or 70% (611/871) of all Swiss ICU beds. Coverage was higher for south-western (203/237; 86%) than for eastern Switzerland (408/634; 64%) (p < 0.001). For the year 2018, sampling density was 10.2 samples per represented ICU bed; sampling density was higher for ICU from the south-west (12.2 per represented bed) compared with those from the east (9.3 per represented bed) (p = 0.002) (Figure 1).

The map is limited to institutions registered in 2018. Circle sizes represent number of samples. Coverage (i.e. number of beds of all registered beds) and sampling density (i.e. number of microbiological samples per represented beds) are given by geographical region.

### Antimicrobial resistance

**Figure 2:**
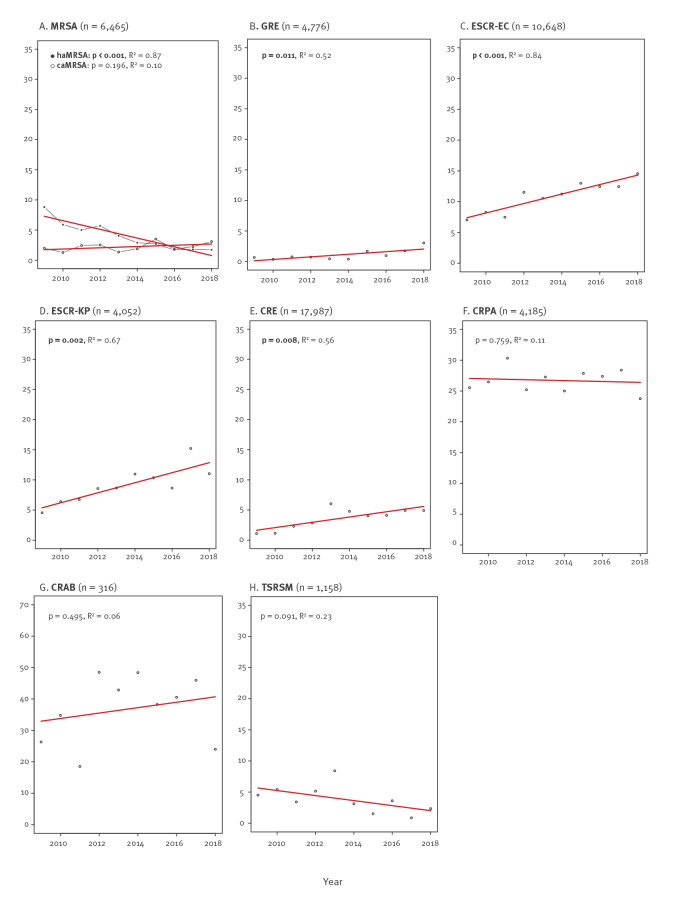
Trends in resistant pathogens in intensive care, Switzerland, 2009–2018 (n = 34,887)

After removal of duplicates, we identified 34,887 tested pathogens. A majority of isolates (n = 19,571; 56%) originated from eastern and 15,316 (44%) from south-western Switzerland. Resistance data by geographical region and year (2009 and 2018) are shown in Table 1; temporal trends of bacterial resistance are shown in Figure 2 (and in Supplementary Figures S2/S3 for eastern and south-western Switzerland separately).

**Table 1:** Number of resistant and total number of isolates from intensive care units, by pathogen, eastern vs south-western Switzerland, 2009 (n = 3,187) and 2018 (n = 4,030)

	2009			2018		
	East	South-west	p value	East	South-west	p value
	n/all	n/all		n/all	n/all	
MRSA	9/292	87/400	**< 0.001**	22/388	20/263	0.410
GRE	1/132	1/168	> 0.999*	14/333	5/293	0.113
ESCR-EC	31/411	35/528	0.678	114/730	65/500	0.232
ESCR-KP	11/139	7/157	> 0.999	34/280	18/171	0.439
CRE	12/833	5/729	0.234	81/1,324	26/851	**0.002**
*Enterobacter* spp.	11/12	5/5	Nd	64/81	18/26	Nd
*Klebsiella pneumoniae*	0/12	0/5	Nd	9/81	3/26	Nd
*Escherichia coli*	0/12	0/5	Nd	5/81	4/26	Nd
Other	1/12	0/5	Nd	3/81	1/26	Nd
CRPA	53/253	65/209	**0.017**	62/254	39/171	0.792
CRAB	7/21	3/17	0.460*	3/17	3/8	0.344*
TSRSM	4/65	2/68	0.434*	2/71	1/57	> 0.999*

CRAB: carbapenem-resistant *Acinetobacter baumannii* complex; CRE: carbapenem-resistant Enterobacterales; CRPA: carbapenem-resistant *Pseudomonas aeruginosa*; GRE: glycopeptid-resistant *Enterococcus faecalis/faecium*; ESCR-EC: extended-spectrum cephalosporin-resistant *Escherichia coli*; ESCR-KP: extended-spectrum cephalosporin-resistant *Klebsiella pneumoniae*; MRSA: meticillin-resistant *Staphylococcus aureus*; Nd: not done; TSRSM: trimethoprim-sulfamethoxazole-resistant *Stenotrophomonas maltophilia.*

Please note that *E. coli* and *K. pneumoniae* isolates were used for both the analysis on ESCR and carbapenem resistance. p values (chi-squared or *Fisher's exact test ) ≤ 0.05 were considered statistically significant (in bold).

caMRSA: community-acquired MRSA; CRAB: carbapenem-resistant *Acinetobacter baumannii* complex; CRE: carbapenem-resistant Enterobacterales; CRPA: carbapenem-resistant *Pseudomonas aeruginosa*; GRE: glycopeptid-resistant *Enterococcus faecalis/faecium*; ESCR-EC: extended-spectrum cephalosporin-resistant *Escherichia coli*; ESCR-KP: extended-spectrum cephalosporin-resistant *Klebsiella pneumoniae*; haMRSA: hospital-associated MRSA; MRSA: meticillin-resistant *Staphylococcus aureus*; TSRSM: trimethoprim-sulfamethoxazole-resistant *Stenotrophomonas maltophilia.*

Y axes show % of resistant (note the different scale for the y axis in panel G). Please note that *E. coli *and *K. pneumoniae* isolates were used for both the analysis on ESCR and carbapenem resistance. The number of all isolates (sensitive and resistant) is indicated in brackets (n). P values ≤ 0.05 were considered statistically significant (in bold).

Among the *S. aureus* isolates (n = 6,465; median: 654/year; range: 530–732), meticillin resistance decreased from 14% to 6% (p = 0.005) from 2009 to 2018. This effect was driven by a decline in hospital-associated MRSA (haMRSA) in south-western Switzerland from 18% to 2% (p < 0.001); the proportion of caMRSA was stable around 3% (p = 0.196). Among the 4,776 enterococcal isolates (median: 478/year; range: 282–627), GRE increased from 1% to 3% (p = 0.011), which was exclusively due to an increase in eastern Switzerland.

We identified 10,648 *E. coli* (median: 1,019/year: range: 817–1,317) and 4,052 *K. pneumoniae* (median: 394/year; range: 328–471) isolates. From 2008 to 2019, ESCR for *E. coli* increased from 7% to 15% (p < 0.001) and for *K. pneumoniae* from 5% to 11% (p = 0.002). Proportions and trends for ESCR were similar between the two geographical regions. The CRE showed an increase from 1% to 5% (p = 0.008; total isolates: n = 17,987) and were more common in eastern Switzerland (5% vs 3%, p < 0.001). In 2009, 16 of 17 CRE were *Enterobacter* spp., whereas in 2018, 77% (82/107) were *Enterobacter* spp., 11% (12/107) were *K. pneumoniae*, and 8% (9/107) were *E. coli*. Among 4,185 *P. aeruginosa* isolates (median: 414/year; range: 381–465), 27% were CRPA, with no change over time (p = 0.759). Isolates of the *A. baumannii* complex were rarely detected (n = 316); 37% of these isolates were CRAB, without temporal change (p = 0.495). Also for the 1,158 *S. maltophilia* isolates, of which 4% were TSRSM, no trend could be observed (p = 0.091).

### Antibiotic consumption

**Figure 3:**
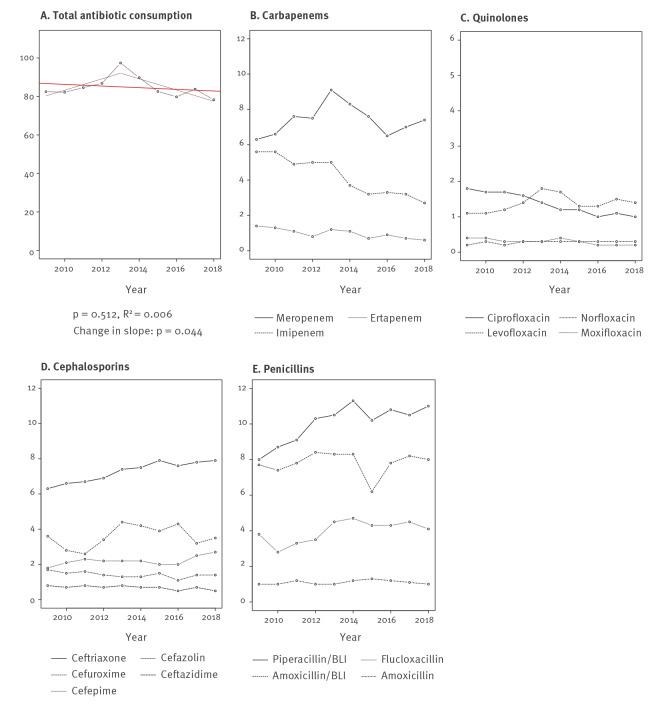
Trends in overall antibiotic consumption and consumption of preselected antibiotic substances in intensive care, in defined daily doses per 100 bed-days, Switzerland, 2009–2018

BD: bed-days; BLI: beta-lactamase inhibitor; DDD: defined daily doses.

Among 35 ICU from south-western and 23 from eastern Switzerland, penicillins, cephalosporins and carbapenems were the three antibiotic groups used the most. For 2018, they accounted for 68% (52.9 DDD/100 BD) of the total (78.3 DDD/100 BD) antibiotic consumption (for a detailed list see Supplementary Table S2). Temporal trends of these antibiotic groups, for quinolones and for the total antibiotic consumption are shown in Figure 3 (and in Supplementary Figures S4/S5 stratified by geographical region). We observed an increase in total antibiotic consumption from 2009 (82.5 DDD/100 BD) to 2013 (97.4 DDD/100 BD), followed by a decrease until 2018 (78.3 DDD/100 BD). Segmented linear regression (performed post-hoc) confirmed these opposing trends (p = 0.044). Similar patterns were observed for the 'watch and reserve' group of antibiotics (WHO AWaRe classification) [15], as well as for carbapenems and quinolones. Consumption of piperacillin/tazobactam increased from 8.0 to 11.0 DDD per 100 BD (p = 0.003) from 2009 to 2018; ceftriaxone consumption increased from 6.3 to 7.9 DDD per 100 BD (p < 0.001). Notable geographical differences were a lower carbapenem (11.4 vs 15.6 DDD/100 BD; p = 0.002) and quinolone consumption (3.0 vs 4.0 DDD/100 BD; p = 0.014) in eastern compared with south-western Switzerland (Supplementary Table S3).

Y axes show DDD per 100 bed-days (note the different scale for the y axis in panels A and C).

### Risk factors for carbapenem resistance

For CRE, institutional antibiotic consumption was significantly (but not very strongly) associated with carbapenem resistance in the mixed effects model (odds ratio (OR) per DDD: 1.01; 95% confidence interval (CI): 1.0–1.02; p = 0.004), as was the year of detection (OR per year: 1.1; 95% CI: 1.1–1.2; p < 0.001). For CRPA, the association with antibiotic consumption was not statistically significant (OR: 1.00; 95% CI: 0.99–1.01; p = 0.083). Interestingly, male sex was positively (OR: 1.3; 95% CI: 1.1–1.6; p = 0.002) and year of detection - although with very small effect - negatively (OR: 0.99; 95% CI: 0.98–0.99; p < 0.001) associated with CRPA. Older age and detection in urine were negatively associated with both CRE and CRPA. Neither geographical region nor university hospital status was predictive for CRE or CRPA (Table 2).

**Table 2:** Factors associated with carbapenem-resistant *Enterobacterales* spp. (n = 14,479) and carbapenem-resistant *Pseudomonas aeruginosa* (n = 3,512) in intensive care, Switzerland, 2009–2018

Covariables	Sensitive		Resistant		Univariable		Multivariable	
	n	%	n	%	OR (95% CI)	p value	OR (95% CI)	p value
*Enterobacterales* spp.	**n = 13,884**		**n = 595**					
Year								
OR per year	Nd	Nd	Nd	Nd	**1.13 (1.13–1.14)**	**< 0.001**	**1.12 (1.10–1.12)**	**< 0.001**
Age								
15– <60	4,046	29	207	35	Ref	Ref	Ref	Ref
60–75	7,440	54	321	54	0.9 (0.8–1.1)	0.539	0.9 (0.8–1.1)	0.441
> 75	2,398	17	67	11	**0.6 (0.5–0.8)**	**0.002**	**0.7 (0.5–0.9)**	**0.006**
Sex								
Female	5,284	38	189	32	Ref	Ref	Ref	Ref
Male	8,600	62	406	68	**1.3 (1.1–1.6)**	**0.001**	1.0 (0.8–1.2)	0.838
Sample localisation								
Blood culture	1,294	9	47	8	Ref	Ref	Ref	Ref
Respiratory sample	6,854	49	349	59	1.2 (0.9–1.7)	0.194	1.3 (0.9–1.7)	0.146
Urine	3,360	24	60	10	**0.4 (0.3–0.6)**	**< 0.001**	**0.4 (0.3–0.6)**	**< 0.001**
Other	2,376	17	139	23	**1.8 (1.3–2.5)**	**0.001**	**1.8 (1.3–2.5)**	**0.001**
Region								
Eastern Switzerland	7,577	55	407	68	Ref	Ref	Ref	Ref
South-western Switzerland	6,307	45	188	32	1.1 (0.6–2.3)	0.717	1.2 (0.6–2.4)	0.570
University status								
Non-university hospital	6,848	49	200	34	Ref	Ref	Ref	Ref
University hospital	7,036	51	395	66	1.1 (0.5–2.5)	0.834	0.9 (0.6–2.1)	0.842
Total antibiotic consumption								
DDD/100BD, median (IQR)	98	87; 116	115	91; 142	**1.02 (1.01–1.02)**	**< 0.001**	**1.01 (1.00–1.02)**	**0.004**
*Pseudomonas aeruginosa*	**n = 2,521**		**n = 991**					
Year								
OR per year	Nd	Nd	Nd	Nd	0.98 (0.92–1.04)	0.502	**0.99 (0.98–0.99)**	**< 0.001**
Age								
15– <60	641	25	400	40	Ref	Ref	Ref	Ref
60–75	1,447	57	513	52	**0.6 (0.5–0.7)**	**< 0.001**	**0.6 (0.5–0.7)**	**< 0.001**
> 75	433	17	78	8	**0.3 (0.2–0.4)**	**< 0.001**	**0.4 (0.3–0.5)**	**< 0.001**
Sex								
Female	876	35	286	29	Ref	Ref	Ref	Ref
Male	1,645	65	705	71	**1.3 (1.1–1.6)**	**< 0.001**	**1.3 (1.1–1.6)**	**0.002**
Sample localisation								
Blood	128	5	54	5	Ref	Ref	Ref	Ref
Respiratory	1,546	61	663	67	1.1 (0.8–1.5)	0.741	1.0 (0.7–1.5)	0.812
Urine	471	19	87	9	**0.5 (0.3–0.7)**	**< 0.001**	**0.5 (0.3–0.8)**	**< 0.001**
Other	376	15	187	19	1.2 (0.8–1.7)	0.384	1.2 (0.8–1.7)	0.377
Region								
Eastern Switzerland	1,472	58	512	52	Ref	Ref	Ref	Ref
South-western Switzerland	1,049	42	479	48	1.4 (0.9–2.2)	0.086	1.4 (0.9–2.0)	0.057
University status								
Non-university hospital	1,193	47	355	36	Ref	Ref	Ref	Ref
University hospital	1,328	53	636	64	1.6 (1.0–2.5)	0.060	1.3 (0.8–1.9)	0.262
Total antibiotic consumption								
DDD/100BD, median (IQR)	98	86; 115	105	89; 125	1.00 (0.99–1.01)	0.205	1.00 (0.99–1.01)	0.083

CI: confidence interval; DDD/100BD: defined daily dose per 100 bed-days; IQR: interquartile range; Nd: not done; OR: odds ratio; Ref: reference group.

p values (mixed effects model) ≤ 0.05 were considered statistically significant (in bold).

### Sensitivity analyses for temporal trends

Considering only those ICU that provided data for at least 8 years between 2009 and 2018, we observed the same temporal trends regarding AMR (in total 28 ICU; 15 from the south-west, 13 from the east of Switzerland) and antibiotic consumption (in total 29 ICU; 14 from the south-west, 15 from the east of Switzerland) (data not shown).

## Discussion

In this laboratory-based analysis of national surveillance data from Swiss ICU, we have shown that the occurrence of MRSA has been decreasing steadily, whereas resistant Gram-negative bacteria including ESCR-EC, ESCR-KP and – of particular concern – CRE have become more frequent between 2009 and 2018. The only modifiable factor associated with CRE was institutional antibiotic consumption. On a national level, antibiotic consumption peaked in 2013 and has since been declining. The strengths of this study were its large sample size, the representativeness of the database for the Swiss ICU setting and the robustness of temporal trends in sensitivity analyses.

Our main finding was the emergence of resistance among *Enterobacterales* in Swiss ICU over the last decade. These data are in line with trends described from other European countries, although the different observation periods overlap only partly with our analysis. In German ICU, ESCR-EC increased from 1.3% to 16.3% and imipenem-resistant *K. pneumoniae* from 0.4% to 1.6% between 2001 and 2015 [16]. In France, ESBL-producing *Enterobacterales* increased by 73% between 2009 and 2013, with higher rates in ICU [17]. In Italy, bloodstream infections caused by CPE, mainly originating in ICU, increased steadily between 2014 and 2017 [18]. Resistant Gram-negative pathogens are also increasing in Swiss non-ICU settings, with up to 22% of *E. coli* being ESCR-EC in long-term care institutions [19,20].

The emergence of CRE, associated with increased morbidity and mortality, is particularly worrying [21]. *Enterobacter* spp. were the predominant species among our CRE. Carbapenem-resistance in *Enterobacter* spp. can be conferred either by de-repressed AmpC in combination with porin loss/modification or by carbapenemase production [22,23]. De-repression of AmpC in *Enterobacter* spp. is usually observed after beta-lactam exposure, which is in accordance with our finding that institutional antibiotic consumption was associated - although not very strongly - with detection of CRE. However, we cannot exclude the possibility that the rise in carbapenem-resistant *Enterobacter* spp. may be due to dissemination of high-risk clones such as ST171 and ST78, which can also carry carbapenemase-encoding plasmids [23]. Another argument suggesting the emergence of CPE in Swiss ICU is the fact that antibiotic consumption, including carbapenem and quinolone consumption, is declining and that *K. pneumoniae* and *E. coli* are increasing among CRE. Increased importation of CPE from high-risk countries or even acquisition in local hospitals could account for their spread, as CPE are considered to spread primarily through horizontal transmission in the healthcare setting [8,24,25]. For instance, the hospital water environment (including drains and sinks) has increasingly been recognised as a reservoir for outbreaks of carbapenem-resistant organisms in the ICU setting [26]. Also, national surveillance data from Switzerland (inpatients and outpatients) show increasing numbers of CPE [27].

We found 27% of *P. aeruginosa* isolates to be carbapenem-resistant. This figure is comparable to data from 36 ICU in the United States which showed 35% CRPA, mostly from the respiratory tract [28]. The association between institutional antibiotic consumption and detection of CRPA was not significant in the multivariable analysis. Indeed, previous studies have shown that total antibiotic consumption is less predictive of CRPA than the consumption of broad-spectrum antibiotics such as carbapenems [5]. In a recent study from selected ICU in Serbia, conducted between 2014 and 2018, overall antibiotic consumption has been steadily decreasing, whereas the occurrence of CRAB and CRE increased [29]. On the other hand, in a before–after study from a French ICU, a reduction in quinolone and carbapenem use was associated with a decrease in CRPA, although total antibiotic use increased [30]. On the individual patient level, previous carbapenem exposure has also been identified as risk factor for CRPA [31].

A favourable trend was observed regarding MRSA in Swiss ICU. The fact that the decline in MRSA was exclusively attributable to haMRSA (and not to caMRSA) has been observed in other countries and has partly been attributed to increased awareness and infection control interventions, including the Dutch search-and-destroy approach [32]. Molecular typing is not routinely performed in Switzerland; this would allow to better discriminate haMRSA from caMRSA. In this context it is important to note that the concept of distinguishing haMRSA and caMRSA has been challenged in recent years, with traditional caMRSA such as USA300 increasingly detected in the healthcare setting and even showing a multi-resistant phenotype [33]. In contrast to MRSA, GRE have become more frequent, particularly in ICU from eastern Switzerland. This signal most probably reflects an interregional VRE outbreak caused by ST796 detected in 2017 [34].

Geographical differences have to be taken into account when explaining AMR trends in Switzerland. Historically, the south-western part of the country has been more affected by AMR than the east [10]. However, our data suggest that these differences are more and more levelling out. Not only were MRSA rates in 2018 very similar between the two regions, but CRE and also GRE were more common in the east than in the south-west. In addition, ICU in the east provided fewer bacterial isolates per represented bed than in the south-west (i.e. lower sampling density), which presumably led to further underestimation of resistance rates in the east. Antibiotic consumption was also somewhat lower in the south-west compared with the east for the year 2018. The reasons for this paradigm shift remain elusive. One (speculative) argumentation is that these favourable trends are a result of enhanced infection control measures implemented during the MRSA epidemic in south-western Switzerland.

Antibiotic consumption in Switzerland has traditionally been lower compared with other European countries [11]. Our data show that antibiotic consumption increased in Swiss ICU from 2009 to 2013. Similarly, data collected from German ICU between 2001 and 2015 have shown a steady increase in antibiotic consumption along with the above-mentioned increase in resistant Gram-negative bacteria [16]. However, consumption in Swiss ICU, including use of antibiotic substances on the 'watch' group of the WHO's AWaRe classification, declined between 2013 and 2018. Likewise, in countries of the European Union and the European Economic Area, overall use of cephalosporins, quinolones and carbapenems has been stabilising or even declining in the same time period [35].

Despite the decreased antibiotic use in intensive care, many antimicrobial stewardship activities are still lacking in a majority of Swiss hospitals [36]. Given the importance of rational antibiotic consumption in the control of AMR, the Swiss National Center for Infection Control launched an initiative to promote antimicrobial stewardship programmes (ASP) in Swiss hospitals in 2016 [37]. The aim was, among others things, to formulate requirements for institutional ASP and facilitate surveillance of antibiotic consumption and antibiotic resistance data using the ANRESIS database. In addition to ASP, particularly in light of the increasing numbers of resistant Gram-negative pathogens, we advocate for a multimodal infection control strategy to monitor and control the spread of AMR in ICU patients, including intensified admission screening programmes along with systematic molecular sequencing of pathogens, contact isolation of colonised or infected patients, strict adherence to standard precautions such as correct hand hygiene and use of personal protective equipment, and alertness to search and destroy environmental sources such as contaminated sink drainage systems, if necessary. We would like to emphasise that ASP (and also infection control measures) should not be reduced to human healthcare; a One Health approach should be pursued considering the complex interplay between human, animal and environmental health. Examples include the use of avoparcin as animal growth promoter in the 1990s, which may have contributed to the spread of glycopeptide-resistant genetic elements in humans [38], or the recent detection of carbapenemase-producing *E. coli* among employees of a Swiss veterinary clinic, which was probably due to cross-transmission from hospitalised companion animals [39].

An important limitation of this study was the lack of information on day of patient admission to the ICU. Although we excluded screening samples from the analysis, part of our data reflect antibiotic susceptibilities of samples collected at the time of ICU admission. This means that some resistant pathogens in our dataset were in fact community-acquired and were not a consequence of high institutional antibiotic consumption. We also suppose that restriction to bacterial samples collected 48 h after ICU admission would have resulted in even higher resistance rates. Furthermore, we could not distinguish between pathogens representing true infection and those only colonising. Also, because of the institution-specific patient identifier, we cannot exclude the possibility of duplicate isolates for patients being admitted to more than one ICU in the same year. Another limitation was the lack of information on individual antibiotic treatment before admission. This prevented us from including antibiotic consumption as a patient level variable in our hierarchical model.

## Conclusion

Resistance among *Enterobacterales* in Swiss ICU is increasing. The rise in carbapenem resistance calls for a multimodal approach to improve surveillance (including systematic screening of ICU patients and molecular typing of resistant isolates) and adherence to standard infection control measures. Although antibiotic consumption is decreasing, the observed association between total antibiotic consumption and the occurrence of CRE underscores the need for antimicrobial stewardship in the Swiss intensive care setting.

## Supplementary Data

793000 Supplement
