# Supplementary material for: Antimicrobial resistance and antibiotic consumption in intensive care units, Switzerland, 2009 to 2018
Source: Euro Surveill. 2021 Nov 18;26(46):2001537. doi: 10.2807/1560-7917.ES.2021.26.46.2001537 (PMC8603405; doi:10.2807/1560-7917.ES.2021.26.46.2001537)
Supplement: Supplement [file 20-01537_BARNSTEINER_AMR_supplement.pdf]

SUPPLEMENT

This supplementary material is hosted by *Eurosurveillance* as supporting information alongside the article “Antimicrobial Resistance and Antibiotic Consumption in Swiss Intensive Care Units from 2009 to 2018”, on behalf of the authors, who remain responsible for the accuracy and appropriateness of the content. The same standards for ethics, copyright, attributions and permissions as for the article apply. Supplements are not edited by *Eurosurveillance* and the journal is not responsible for the maintenance of any links or email addresses provided therein.

SUPPLEMENTARY TABLES

Supplementary Table S1. Swiss intensive care units (ICUs) (n=41) included for analysis of representativeness for the year 2018. All ICUs are interdisciplinary departments - only the cantonal hospitals of Aarau and St. Gallen have separately managed medical and surgical ICUs.

Hospital	*City*	*Region*	*Beds*	*Beddays*
**University hospitals**				
Universitätsspital Basel	Basel	East	46	11,934
Inselspital	Bern	East	39	9,405
Hôpitaux Universitaires de Genève	Genève	South-West	34	10,659
Centre hospitalier universitaire vaudois	Lausanne	South-West	35	11,160
UniversitätsSpital	Zürich	East	64	23,605
**Non-university hospital**s				
Kantonsspital Aarau	Aarau	East	24	8,050
Ospedale San Giovanni	Bellinzona	South-West	9	2,771
Hirslanden Klinik Beau-Site	Bern	East	12	3,309
Spital Tiefenau Spitalnetz Bern	Bern	East	8	1,794
Lindenhofspital	Bern	East	8	na*
Kantonsspital Graubünden	Chur	East	11	1,981
Hôpital du Jura - site de Delémont	Delémont	South-West	6	na
Spital Thurgau AG	Frauenfeld	East	9	6,732
Hôpital Fribourgeois - Site Fribourg	Fribourg	South-West	19	5,108
Spitalregion RWS - Spital Grabs	Grabs	East	6	na
Ospedale Regionale di Locarno La Carità	Locarno	South-West	7	2,256
Ospedale Regionale di Lugano, Sede Civico	Lugano	South-West	12	3,972
Cardiocentro Ticino	Lugano	South-West	9	na
Clinica Luganese	Lugano	South-West	6	na
Luzerner Kantonsspital	Luzern	East	22	6,691
Ospedale Regionale della Beata Vergine	Mendrisio	South-West	6	1,715
Hôpital du Chablais-Aigle	Monthey	South-West	7	1,696
Kantonsspital Münsterlingen	Münsterlingen	East	12	na
Hôpital Neuchâtelois Pourtalès	Neuchâtel	South-West	10	na
Kantonsspital Olten	Olten	East	9	2,083
Kantonsspital Schaffhausen	Schaffhausen	East	8	2,160
Centre Hospitalier du Centre du Valais	Sion	South-West	14	4,030
Bürgerspital Solothurn	Solothurn	East	9	2,234
Kantonsspital St.Gallen	St. Gallen	East	42	9,418
Hirslanden Klinik Stephanshorn	St. Gallen	East	6	na
Kantonsspital Nidwalden	Stans	East	6	1,640
Spital Sursee-Wolhusen	Sursee	East	6	781
Hôpital Riviera Site du Samaritain	Vevey	South-West	7	1,830
Spitalzentrum Oberwallis	Visp	South-West	6	1,027
Kantonsspital Winterthur	Winterthur	East	18	4,451
Luzerner Kantonsspital Wolhusen	Wolhusen	East	6	785
Etablissements Hospitaliers du Nord Vaudois	Yverdon-les-Bains	South-West	8	1,985
Spital Zollikerberg	Zollikerberg	East	7	na
Klinik Hirslanden	Zürich	East	22	5,179
Hirslanden Klinik im Park	Zürich	East	8	na

***na, not available.**

Supplementary Table S2. Consumption of antibiotic classes including selected antibiotic substances and total annual antibiotic consumption in Swiss intensive care units expressed in DDD/100 bed-days.

	2009	*2010*	*2011*	*2012*	*2013*	*2014*	*2015*	*2016*	*2017*	*2018*	*P*, R^2
**Penicillins**	**22.6**	**21.8**	**23.3**	**25.5**	**26.3**	**27.2**	**23.2**	**25.6**	**26.1**	**25.7**	**0.048, .33**
Piperacillin/BLI[1]	8.0	8.7	9.1	10.3	10.5	11.3	10.2	10.8	10.5	11.0	**0.003, .65**
Amoxicillin/BLI[1]	7.7	7.4	7.8	8.4	8.3	8.3	6.2	7.8	8.2	8.0	0.895, .12
Flucloxacillin	3.8	2.8	3.3	3.5	4.5	4.7	4.3	4.3	4.5	4.1	**0.036, .37**
Amoxicillin	1.0	1.0	1.2	1.0	1.0	1.2	1.3	1.2	1.1	1.0	0.423, .03
**Cephalosporins**	**19.9**	**18.6**	**18.1**	**19.1**	**19.3**	**18.0**	**18.9**	**20.0**	**18.4**	**16.5**	0.207, .09
Ceftriaxone	6.3	6.6	6.7	6.9	7.4	7.5	7.9	7.6	7.8	7.9	**<0.001, .89**
Cefuroxime	3.6	2.8	2.6	3.4	4.4	4.2	3.9	4.3	3.2	3.5	0.324, .01
Cefepime	1.8	2.1	2.3	2.2	2.2	2.2	2.0	2.0	2.5	2.7	0.053, .32
Cefazolin	1.7	1.5	1.6	1.4	1.3	1.3	1.5	1.1	1.4	1.4	0.062, .29
Ceftazidime	0.8	0.7	0.8	0.7	0.8	0.7	0.7	0.5	0.7	0.5	**0.020, .45**
**Carbapenems**	**13.3**	**13.5**	**13.6**	**13.3**	**15.3**	**13.1**	**11.5**	**10.7**	**10.9**	**10.7**	**0.012, .51**
Meropenem	6.3	6.6	7.6	7.5	9.1	8.3	7.6	6.5	7.0	7.4	0.693, .10
Imipenem	5.6	5.6	4.9	5.0	5.0	3.7	3.2	3.3	3.2	2.7	**<0.001, .90**
Ertapenem	1.4	1.3	1.1	0.8	1.2	1.1	0.7	0.9	0.7	0.6	**0.002, .68**
**Tetracyclines**	**1.0**	**1.1**	**1.1**	**1.1**	**1.7**	**1.8**	**1.3**	**0.9**	**1.3**	**1.2**	0.614, .08
Doxycycline	0.5	0.5	0.4	0.5	0.7	0.8	0.5	0.5	0.5	0.7	0.324, .01
Tigecyclin	0.4	0.5	0.3	0.5	0.6	0.7	0.4	0.3	0.4	0.3	0.496, .06
**MLSB-family**[2]	**5.3**	**5.7**	**5.5**	**5.9**	**6.1**	**6.4**	**5.8**	**5.9**	**5.4**	**6.3**	0.199, .10
Clarithromycin	2.4	2.5	2.3	2.5	2.8	2.5	2.4	2.5	2.2	2.1	0.230, .07
Erythromycin	1.9	2.1	2.2	2.0	1.9	2.0	1.8	2.1	1.5	2.3	0.720, .11
Clindamycin	0.7	0.7	0.6	0.8	0.9	0.9	0.9	0.9	1.1	1.5	**0.002, .69**
Azithromycin	0.3	0.4	0.4	0.6	0.5	1.0	0.7	0.4	0.6	0.4	0.439, .04
**Aminoglycosides**	**5.2**	**3.1**	**3.7**	**2.6**	**6.3**	**3.3**	**3.0**	**1.5**	**1.7**	**1.9**	0.052, .32
Gentamicin	1.0	1.2	1.1	1.0	1.1	1.0	1.0	0.6	0.8	0.7	**0.008, .56**
Tobramycin	0.6	0.8	1.1	0.8	0.7	1.4	1.4	0.3	0.4	0.4	0.432, .02
Amikacin	0.6	0.5	0.5	0.6	0.7	0.6	0.6	0.6	0.5	0.4	0.392, .04
**Quinolones**	**3.6**	**3.7**	**3.5**	**3.7**	**3.9**	**3.6**	**3.1**	**2.9**	**3.1**	**3.2**	**0.020, .45**
Ciprofloxacin	1.8	1.7	1.7	1.6	1.4	1.2	1.2	1.0	1.1	1.0	**<0.001, .93**
Levofloxacin	1.1	1.1	1.2	1.4	1.8	1.7	1.3	1.3	1.5	1.4	0.206, .09
Norfloxacin	0.4	0.4	0.3	0.3	0.3	0.4	0.3	0.2	0.2	0.2	**0.005, .61**
Moxifloxacin	0.2	0.3	0.2	0.3	0.3	0.3	0.3	0.3	0.3	0.3	0.062, .29
**Glycopeptides**	**4.5**	**5.9**	**6.0**	**5.9**	**6.9**	**6.3**	**6.0**	**4.0**	**5.3**	**3.7**	0.280, .04
Vancomycin	4.1	4.8	5.3	5.2	6.0	6.0	5.4	3.9	5.0	3.5	0.534, .07
Teicoplanin	0.4	1.1	0.7	0.7	0.9	0.3	0.6	0.1	0.3	0.2	**0.046, .34**
**Others**											
Metronidazole	1.6	1.7	1.7	1.8	2.0	1.7	1.5	1.9	1.7	1.6	0.946, .12
TMP/SMX[3]	1.3	1.5	1.9	1.8	2.1	1.6	1.6	1.7	1.6	1.6	0.767, .11
Colistin	0.4	1.0	1.2	1.4	2.3	2.3	1.0	0.5	1.7	0.7	0.830, .12
Aztreonam	0.3	0.1	0.4	0.1	0.2	0.1	0.3	0.1	0.1	0.1	0.190, .11
Fosfomycin	0.0	0.3	0.0	0.1	0.0	0.1	0.1	0.1	0.2	0.1	0.668, .10
Daptomycin	0.5	1.8	2.3	2.4	2.6	1.7	1.9	1.6	2.1	2.4	0.247, .06
Linezolid	0.2	0.2	0.3	0.2	0.2	0.2	0.1	0.1	0.1	0.1	**0.007, .57**
**Total**	**82.5**	**82.2**	**84.6**	**86.8**	**97.4**	**89.8**	**82.6**	**79.8**	**83.8**	**78.3**	0.512, .06
**Watch**[4]	**42.1**	**44.5**	**44.6**	**46.6**	**51.7**	**48.3**	**44.7**	**41.6**	**43.0**	**41.8**	0.555, .07
**Reserve**[5]	**3.8**	**6.0**	**6.8**	**6.9**	**8.1**	**7.3**	**5.9**	**5.4**	**8.1**	**6.9**	0.194, .10

1: BLI, Beta-lactamase inhibitor; 2: MLSB, Macrolide Lincosamide Streptogramin B; 3: TMP/SMX, Trimethoprim/Sulfamethoxazole; 4: Watch group according to AWaRe classification Azithromycin, Cefixime, Cefpodoxime, Ceftazidime, Ceftibuten, Ceftriaxone, Ciprofloxacin, Clarithromycin, Ertapenem, Erythromycin, Imipenem, Levofloxacin, Meropen-em, Moxifloxacin, Norfloxacin, Ofloxacin, Piperacillin/BLI, Polymyxin B, Roxithromycin, Teicoplanin, Ticarcillin/BLI, Vancomycin; 5: Reserve group according to AWaRe classification Aztreonam, Cefepime, Ceftaroline, Ceftobiprole, Ceftolozane/BLI, Colistin, Daptomycin, Fosfomycin, Linezolid, Tedizolid, Tigecyclin; ; P values (linear regression) ≤0.05 were considered statistically significant

Supplementary Table S3. Consumption of antibiotic classes in DDD/100 bed-days in 2009 and 2018 of Eastern *vs.* South-Western Switzerland.

	2009			*2018*		
	East	*South-West*	*P value*	*East*	*South-West*	*P value*
**Penicillins**	22.7	22.7	.379	27.0	23.2	**.008**
**Cephalosporins**	19.6	18.7	.675	16.9	15.9	.266
**Carbapenems**	11.4	17.6	.303	10.4	12.1	.145
**Tetracyclines**	0.9	1.0	.765	1.1	1.2	.931
**MLSB-family**[1]	4.8	6.4	**.023**	6.0	6.8	.116
**Aminoglycosides**	12.4	1.8	**.020**	2.0	1.7	.563
**Quinolones**	3.1	4.5	.053	2.6	4.0	**.007**
**Glycopeptides**	4.0	5.7	**.012**	4.3	2.9	.751
**Others**	6.9	5.5	.189	8.7	8.1	.564
**Total**	85.8	83.9	.465	79.0	75.9	.840
**Watch**[2]	37.2	50.9	<.001	41.8	42.5	.279
**Reserve**[3]	4.7	2.1	.113	7.2	5.9	.427

1: MLSB, Macrolide Lincosamide Streptogramin B; 2: Watch group according to AWaRe classification Azithromycin, Cefixime, Cefpodoxime, Ceftazidime, Ceftibuten, Ceftriaxone, Ciprofloxacin, Clarithromycin, Ertapenem, Erythromycin, Imipenem, Levofloxacin, Meropen-em, Moxifloxacin, Norfloxacin, Ofloxacin, Piperacillin/BLI*, Polymyxin B, Roxithromycin, Teicoplanin, Ticarcillin/BLI*, Vancomycin; 3: Reserve group according to AWaRe classification Aztreonam, Cefepime, Ceftaroline, Ceftobiprole, Ceftolozane/BLI*, Colistin, Daptomycin, Fosfomycin, Linezolid, Tedizolid, Tigecyclin; *: BLI, Beta-lactamase inhibitor; P values (Wilcoxon rank sum test) ≤0.05 were considered statistically significant

SUPPLEMENTARY FIGURES

Supplementary Figure S1. Number of intensive care units (by geographic region) per analysis.

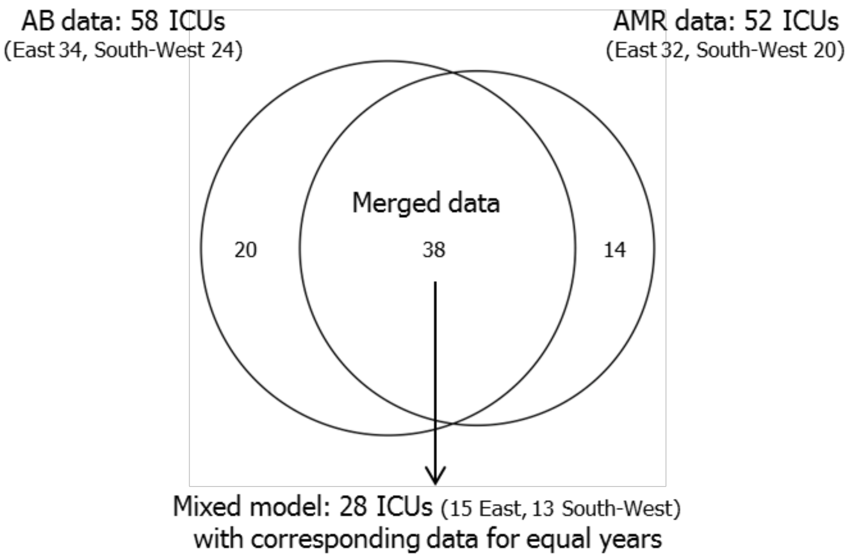

Note: These numbers refer to ICUs included from 2009 to 2018, whereas the 41 ICUs mentioned in paragraph 1 of the results refer to ICUs, for which data were available for the year 2018 (for analysis of representativeness).

Supplementary Figure S2. Trends in resistant pathogens in intensive care units of Eastern Switzerland; number of all isolates (sensitive and resistant) indicated in brackets (n).

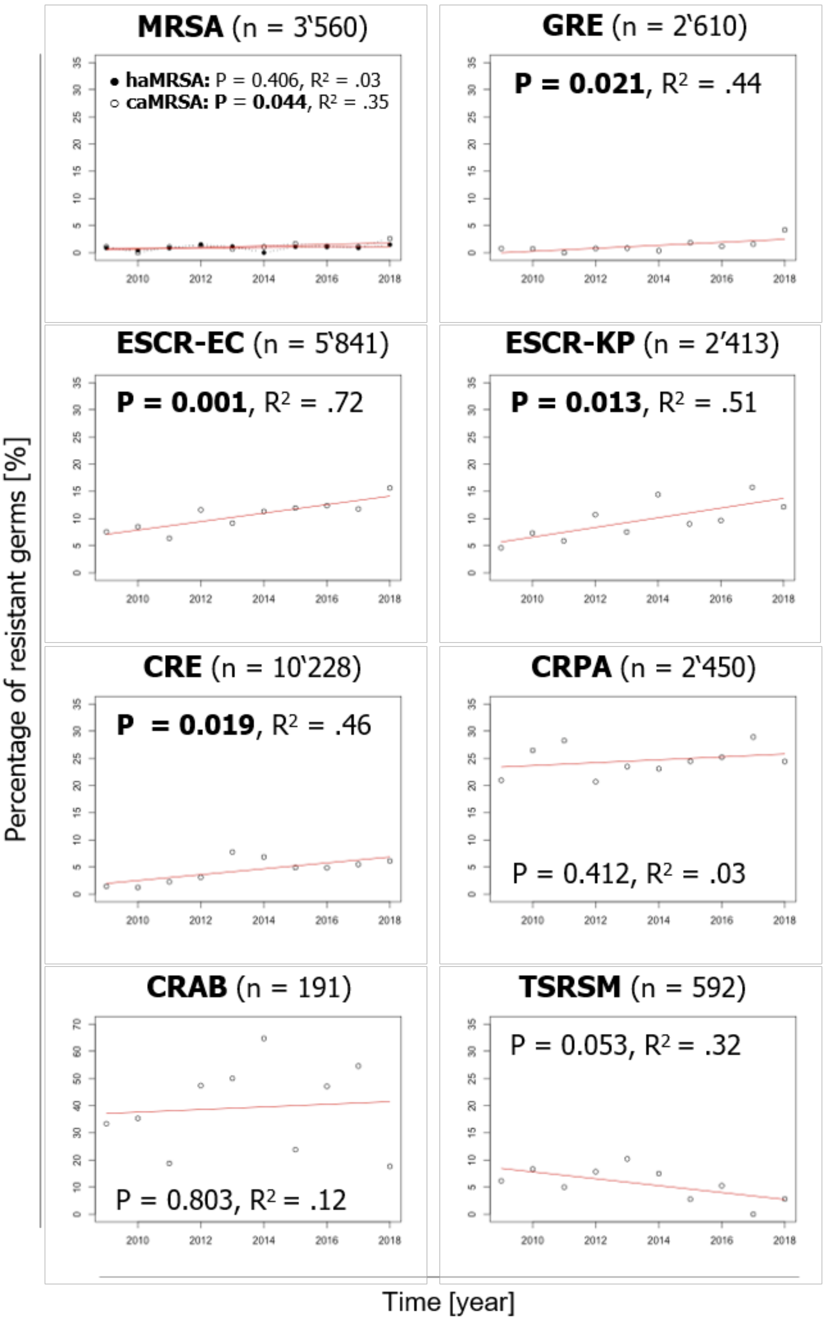

1: MRSA, methicillin-resistant Staphylococcus aureus; ha, hospital-associated; ca, community-associated; 2: GRE, glycopeptid-resistant Enterococcus faecalis/faecium; 3: ESCR-EC, extended-spectrum cephalosporin-resistant Escherichia coli; 4: ESCR-KP, extended-spectrum cephalosporin-resistant Klebsiella pneumoniae; 5: CRE, carbapenem-resistant Enterobacterales; 6: CRPA, carbapenem-resistant Pseudomonas aeruginosa; 7: CRAB, carbapenem-resistant Acinetobacter baumannii complex; 8: TSRSM, trimethoprim-sulfamethoxazole-resistant Stenotrophomonas maltophilia; P values (linear regression) ≤0.05 were considered statistically significant

Supplementary Figure S3. Trends in resistant pathogens in intensive care units of South-Western Switzerland; number of all isolates (sensitive and resistant) indicated in brackets (n).

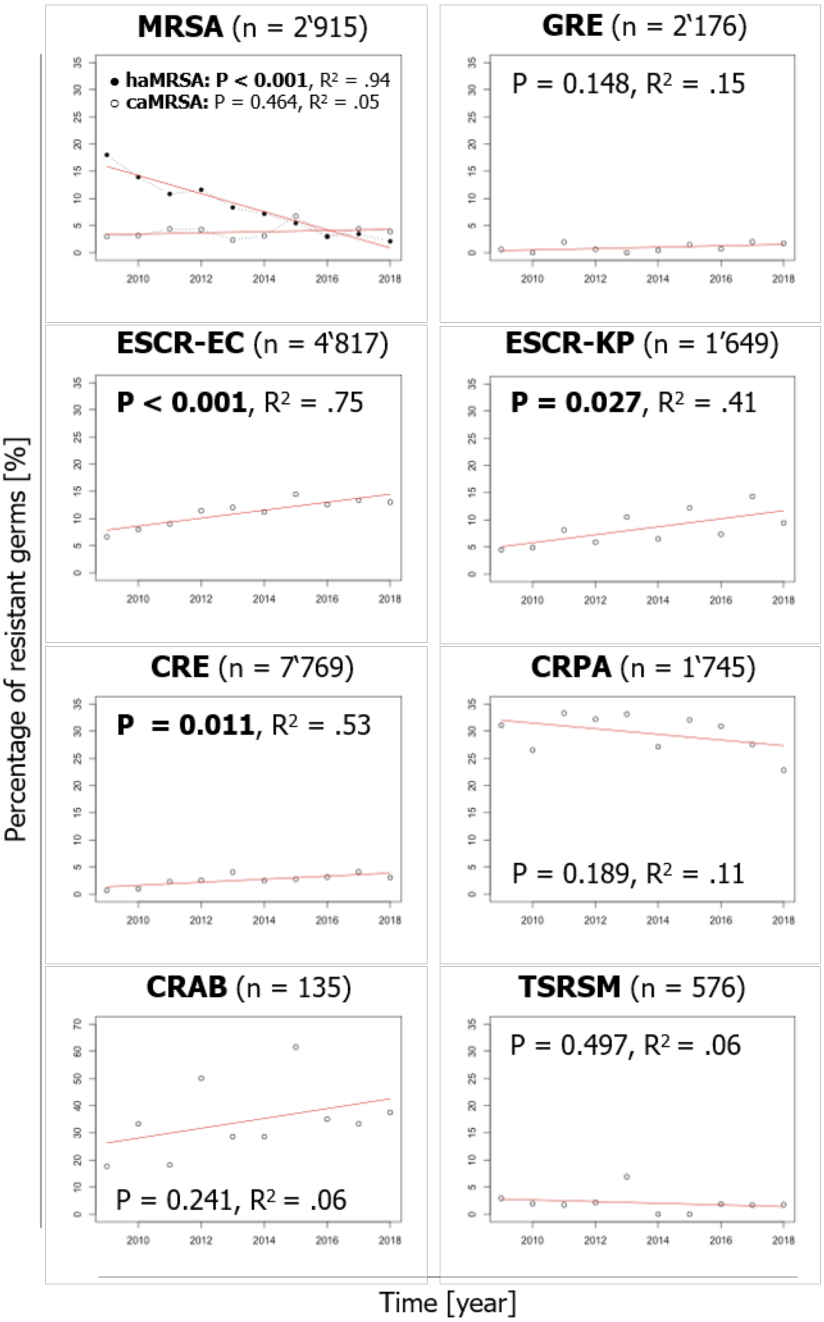

1: MRSA, methicillin-resistant Staphylococcus aureus; ha, hospital-associated; ca, community-associated; 2: GRE, glycopeptid-resistant Enterococcus faecalis/faecium; 3: ESCR-EC, extended-spectrum cephalosporin-resistant Escherichia coli; 4: ESCR-KP, extended-spectrum cephalosporin-resistant Klebsiella pneumoniae; 5: CRE, carbapenem-resistant Enterobacterales; 6: CRPA, carbapenem-resistant Pseudomonas aeruginosa; 7: CRAB, carbapenem-resistant Acinetobacter baumannii complex; 8: TSRSM, trimethoprim-sulfamethoxazole-resistant Stenotrophomonas maltophilia; P values (linear regression) ≤0.05 were considered statistically significant

Supplementary Figure S4. Trends in overall antibiotic consumption and consumption of preselected antibiotic substances in ICUs of Eastern Switzerland expressed in DDD/100 bed-days.

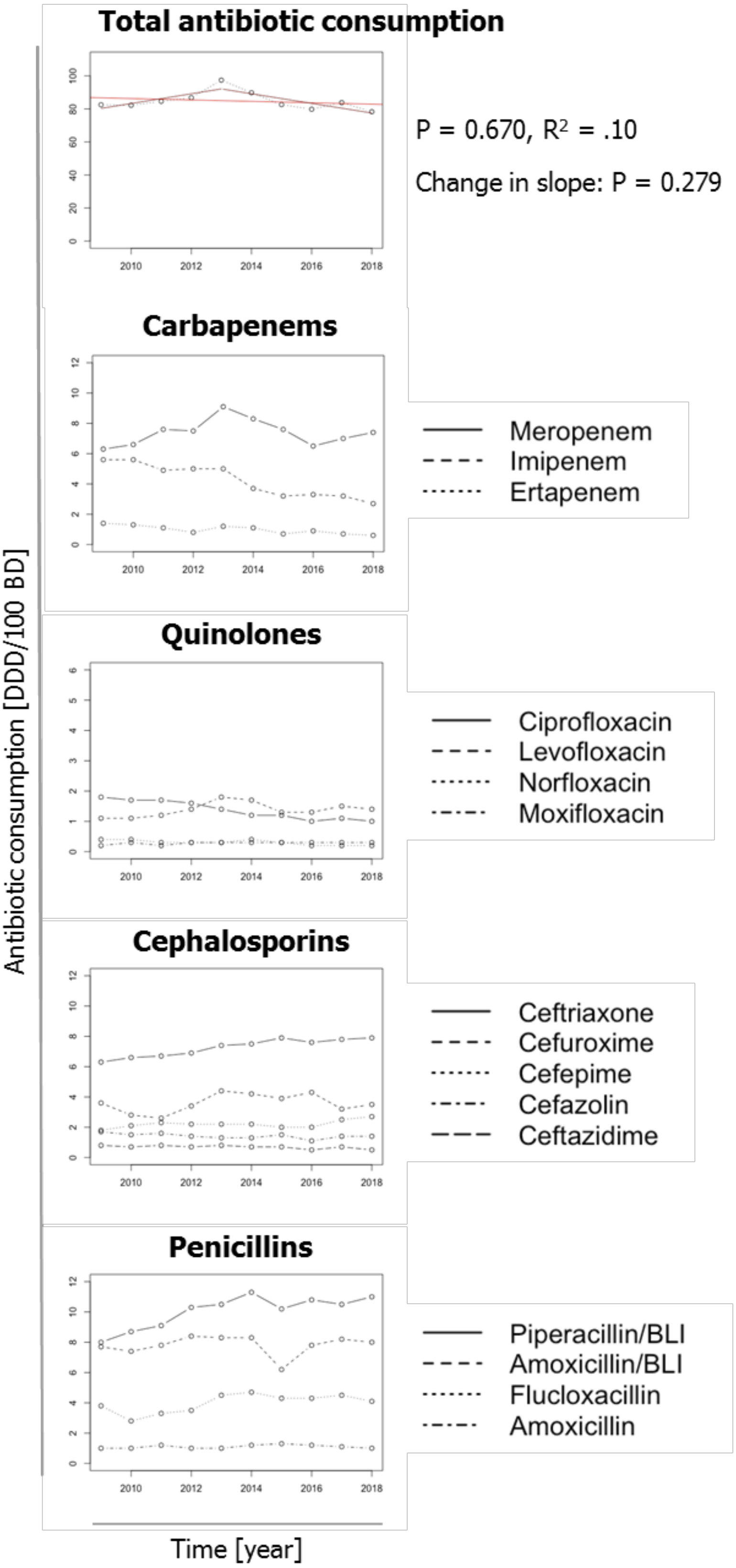

BLI, Beta-lactamase inhibitor; P values (linear regression; segmented linear regression) ≤0.05 were considered statistically significant

Supplementary Figure S5. Trends in overall antibiotic consumption and consumption of preselected antibiotic substances in ICUs of South-Western Switzerland expressed in DDD/100 bed-days.

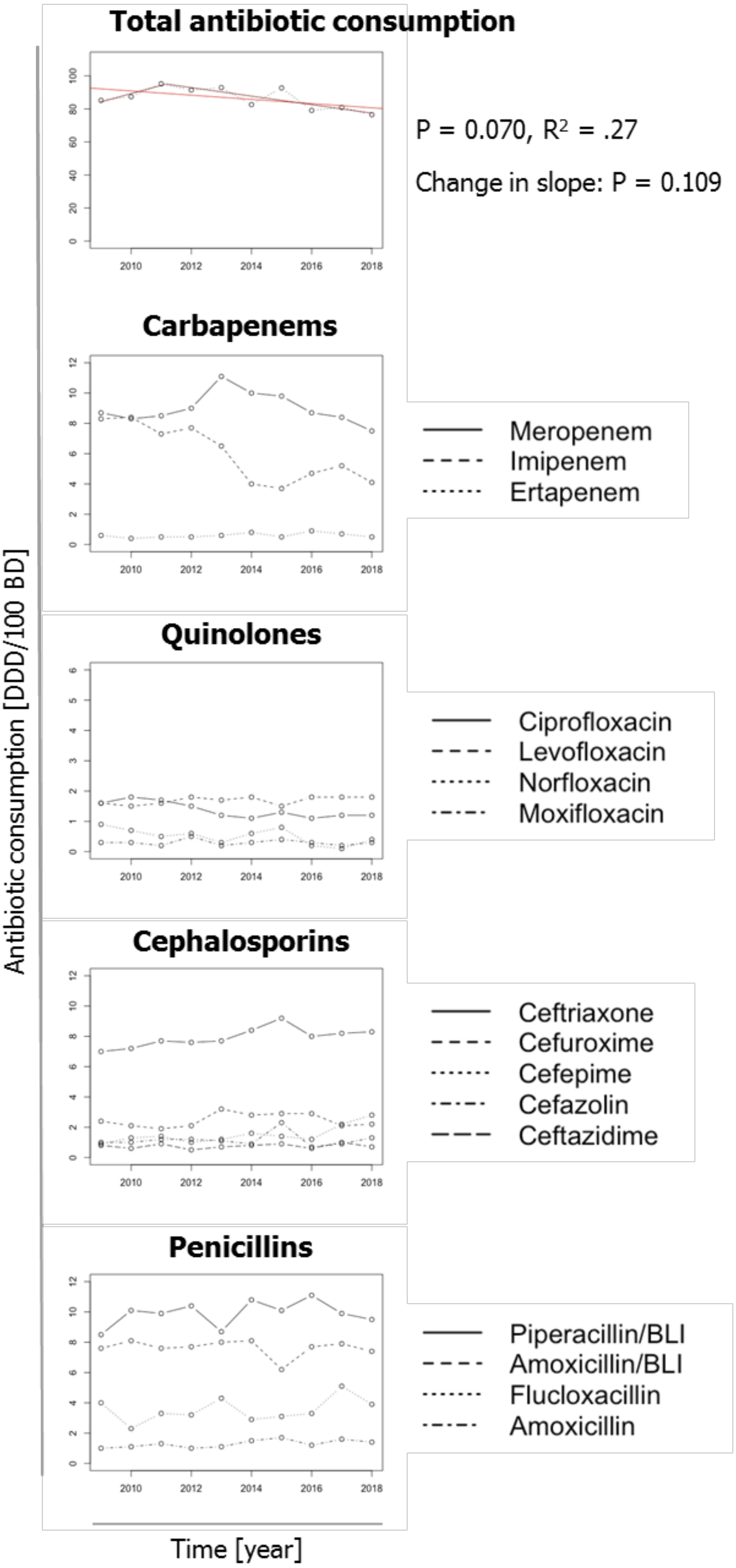

BLI, Beta-lactamase inhibitor; P values (linear regression; segmented linear regression) ≤0.05 were considered statistically significant
